# The Potential Implications of Sex-Specific Differences in the Intestinal Bacteria of the Overwintering Wolf Spider *Pardosa astrigera* (Araneae: Lycosidae)

**DOI:** 10.3390/insects15070490

**Published:** 2024-06-30

**Authors:** Ningkun Li, Quan Yuan, Yaru Qi, Pengfeng Wu, Shuyan Cui, Guo Zheng

**Affiliations:** 1 College of Life Science, Shenyang Normal University, Shenyang 110034, China; 2 Liaoning Key Laboratory for Biological Evolution and Agricultural Ecology, Shenyang 110034, China

**Keywords:** gut microbiome, bacterium, diversity, community composition, overwintering

## Abstract

**Simple Summary:**

There are differences in the cold resistance and anti-freeze compound levels in the two sexes of *Pardosa astrigera*, driving us to explore the differences in their intestinal microbiome. In this study, intestinal bacterial communities of *P*. *astrigera* in winter were compared using 16S rRNA high-throughput sequencing. The results showed significant differences in most alpha diversity indices and the relative abundances of dominant bacteria between males and females. Our research reveals the significant role of sex in shaping the diversity and composition of intestinal bacteria in overwintering *P*. *astrigera*. We suggest that *Pseudomonas versuta* (belonging to Proteobacteria) and *Rhodococcus erythropolis* (belonging to Actinobacteriota) may have the potential to play key roles in overwintering *P. astrigera*.

**Abstract:**

Gut microbiota can promote the resistance of host arthropods to low-temperature stress. Female *Pardosa astrigera* have a lower anti-freeze compound level and weaker resistance to cold temperatures than the males in winter, which implies that their intestinal bacteria may be different during overwintering. This study primarily compared the intestinal bacterial communities between the two sexes of *P*. *astrigera* in a temperate region using 16S rRNA gene sequencing. Our findings indicated that the Chao1 and Shannon indices of intestinal bacteria in females were significantly higher than those in males, while the Simpson index in females was significantly lower than that in males. The male intestinal bacterial community was characterized by Proteobacteria and Actinobacteriota at the phylum level and by *Pseudomonas* and *Rhodococcus* at the genus level, with total relative abundances of 89.58% and 85.22%, respectively, which were also significantly higher than those in females, whose total relative abundances were 47.49% and 43.68%, respectively. In contrast, the total relative abundances of Bacteroidota and Firmicutes were significantly lower in males (4.26% and 4.75%, respectively) than in females (26.25% and 22.31%, respectively). Noteworthy divergences in bacterial communities were also found through an LEfSe analysis between females and males. Additionally, the results of the PICRUSt2 analysis showed that six out of eleven level-2 pathways related to key metabolic functions were significantly (or marginally significantly) higher in females than males, and five other level-2 pathways were significantly (or marginally significantly) lower in females than males. Our results imply that significant gender differences exist in intestinal bacterial communities of overwintering *P*. *astrigera*. We suggest that *Pseudomonas versuta* (belonging to Proteobacteria) and *Rhodococcus erythropolis* (belonging to Actinobacteriota) may have the potential to play key roles in overwintering *P*. *astrigera*.

## 1. Introduction

In temperate regions, shortages of prey and low temperatures during winter inevitably challenge overwintering arthropods. While the survival adaptations of overwintering insects have been well documented [1], there has been little focus on spiders [2]. Spiders are among the most important natural enemies of pests in ecosystems. Understanding overwintering ecology is important not only for the survival of a spider’s local population but also for the health of agroecosystems in the following season, as the number of surviving winter spiders can serve as important biocontrol agents for pest pressure. Some winter studies regarding overwintering spiders have documented a number of important features, such as energy balance and metabolic changes [2], body size and survival [3], the tradeoff between increased mortality and growth [4], intraguild predation [5], protection strategy [6], and predation activity [7] in winter. Microbial communities may significantly influence the varied lifestyles of insect hosts [8] and can enhance the cold tolerance and survival of host arthropods under cold conditions [9]. Only a few studies have focused on intestinal bacteria and their effects on overwintering spiders in temperate or higher latitude regions to date [10,11].

In addition to critical roles in fundamental nutritional processes [8], intestinal bacteria can also increase the tolerance to abiotic challenges such as heat stress [12]. Moghadam et al. [13] reported that feeding flies with low-temperature-cultured intestinal bacteria could increase their tolerance to the cold. Intestinal bacteria were found to be associated with the overwintering process of host insects at low temperatures. For example, Wang et al. [14] and Hou et al. [15] found that gut bacterial communities in beetle larvae showed variation in relative abundance during the overwintering stage. Liu et al. [16], suggested that honeybees maintained their intestinal ecosystem balance and increased the abundance of gut probiotics in response to environmental and nutritional pressures in winter. Finally, Ferguson et al. [17] demonstrated that the seasonal shifts in intestinal microbiota were concurrent with changes in tolerance and immunity to cold temperatures. Furthermore, some researchers have explored the mechanisms by which microbes affect the cold tolerance of insects. Raza et al. [18] suggested that intestinal microbiota promote the host resistance to low-temperature stress in oriental fruit fly by stimulating its arginine and proline metabolism pathway. Fikrig et al. [9] also proved that the bacterium *Anaplasma phagocytophilum* induced the expression of anti-freeze glycoproteins, thereby increasing the cold tolerance and survival of *Ixodes scapularis* ticks. Additionally, Tanaka and Watanabe [10] found that the house spider (*Achaearanea tepidariorum*) fed flies with ice-nucleating active bacteria had those microbes in their gut, and the cold tolerance of the spiders was reduced. Therefore, the intestinal microbiome may be the key to understanding freeze-tolerance in a range of invertebrates [12].

As a dominant ground-dwelling spider in the temperate regions of China, *Pardosa astrigera* L. Koch is an active predator in the terrestrial environment [19]. It is widely distributed in various ecosystems and plays a crucial role as a natural enemy in cropland ecosystems [20]. Studies concerning *P*. *astrigera* have been conducted, such as that by Yang et al. [21], who indicated that the substantially darker and larger bodies of the overwintering generation of *P*. *astrigera* are adaptive to adverse conditions. Tanaka and Ito [6] found that the overwintering *P*. *astrigera* (collected from Sapporo, Japan, at a similar latitude to Shenyang, China) accumulated a high concentration of glycerol and a small proportion of myo-inositol, which were partially related to cold resistance. Interestingly, we found that female *P*. *astrigera* had a lower glycerol accumulation (4505.84 ± 745.96) than male *P*. *astrigera* (5523.54 ± 254.89) (unpublished data), which indicates that males may have greater cold resistance than females. Similarly, Liu [22] demonstrated in a laboratory setting that overwintering female *P*. *astrigera* had an obviously lower survival rate than males at −4 °C. Despite the abovementioned research, no studies concerning the features of the intestinal bacterial communities in the two sexes of overwintering *P*. *astrigera* have been conducted.

The historical lowest temperature in Shenyang, Liaoning Province, China, is below −30 °C, and in most years, weather below −20 °C will last for several days. Thus, it is an ideal area for investigating the effects of low-temperature stress on the intestinal bacteria of *P*. *astrigera*. With the aim to compare the differences in intestinal microbiota between the two sexes of overwintering *P*. *astrigera*, all individuals were collected from a very limited spatial range under natural conditions in the middle of winter in the Shenyang region. Sexual variation has been proven to be a crucial factor in shaping the intestinal bacterial community, and more active female arthropods have been associated with a higher intestinal microbiota diversity [23,24,25,26]. We hypothesize that the diversity and composition of intestinal bacteria will differ between the two sexes and that the functional profiles of these microbiota may relate to the cold tolerance of spiders.

## 2. Materials and Methods

### 2.1. Spider Collection

A total of 60 spiders (30 females and 30 males) were collected by hand from 12 to 14 December 2022 (average temperature was −8.5 °C), from a cornfield beside the Puhe River in Shenbei New District of Shenyang city, China (41°56’ N, 123°22’ E). Due to the low temperatures, the spiders were found to be inactive in cracks of the surface soil. All individuals were collected from a very small range (approximately 10 m^2^) of a highly homogeneous environment. Spiders were kept singly in 5 mL tubes, then brought to the laboratory and kept outside under natural conditions before dissection (average temperature was −12.4 °C).

### 2.2. Intestinal Extraction

All instruments used for dissection and handling were sterilized using an autoclave. The operating table was wiped with 75% alcohol and then exposed to an ultraviolet lamp for 60 min to ensure sterility. Each spider was placed in a tube containing 75% alcohol, gently shaken for 5 min, transferred to a petri dish also filled with 75% alcohol and swiped with a cotton ball 5 to 10 times. Afterwards, the spider was rinsed 3 times with sterile water and transferred to a petri dish filled with sterile phosphate-buffered saline (PBS) to eliminate the residual alcohol. The opisthosoma was dissected using sterilized scissors in PBS to isolate the guts under the microscope, and the entire procedure was conducted on ice. The gut was washed with sterile water, placed at the bottom of a 1.5 mL microcentrifuge tube and temporarily stored in a refrigerator at −20 °C (Haier BCD-252WBCS, Qingdao, China). This process was repeated for 10 individuals, and all 10 guts were placed in the same tube as one sample. The tube was flash froze in liquid nitrogen and stored in a −80 °C refrigerator (AUCMA DW-86L500, Qingdao, China) until DNA extraction. Three biological replicates were obtained from each sex.

### 2.3. DNA Extraction, PCR Amplification, and Sequencing

The total DNA of each sample was extracted using the FastDNA Spin Kit for Soil (MP Biomedicals, Santa Ana, CA, USA), and the collected genomic DNA was assessed via 1% agarose gel electrophoresis. Subsequently, 16S rRNA was amplified via PCR containing 4 μL 5× buffer, 2 μL dNTPs (2.5 mM), 0.8 μL of each primer-specific primer (5 μM) targeting the V3–V4 region of the 16S rRNA gene [27], including forward (338F) 5’-ACTCCTACGGGAGGCAGCAG-3’ and reverse (806R) 5’-GGACTACHVGGGTWTCTAAT-3’, 0.4 μL DNA polymerase, 10 ng template DNA, and ddH_2_O to a final reaction volume of 20 µL. The PCR cycling conditions were applied as follows: initial denaturation at 95 °C for 3 min, followed by 29 cycles of denaturation at 95 °C for 30 s, annealing at 53 °C for 30 s, and extension at 72 °C for 45 s per cycle. A final extension at 72 °C for 10 min was concluded for amplification. The PCR products were extracted via 2% agarose gel and purified using an AxyPrep DNA Gel Extraction Kit (Axygen Biosciences, Union City, CA, USA), according to the manufacturer’s instructions. Quantitative analysis was performed using the QuantuiFluor™-ST (Promega, Solana Beach, CA, USA). Sequencing was carried out on the Illumina MiSeq platform at Majorbio Bio-Pharm Technology Co., Ltd. in Shanghai, China.

### 2.4. Bioinformatics and Sequence Analysis

The following pre-processing steps were implemented on the initial reads obtained from the Illumina MiSeq platform to ensure the generation of more reliable and high-quality sequencing results (valid reads), which were demultiplexed and filtered using FASTP (version 0.19.6) [27,28]. Then, the reads were merged together using FLASH (version 1.2.115) [29]. High-quality reads meeting a minimum of the 97% identity threshold were clustered using UPARSE (version 7.0) [30], while simultaneously eliminating any chimeric sequences to create operational taxonomic units (OTUs). For analysis purposes, only OTUs containing a minimum of 50 reads in total were considered and analyzed via an RDP classifier (version 2.11) [31] against the Silva 16S rRNA database (version 138) [32] with a confidence threshold of 70%.

### 2.5. Statistical Analyses

Mothur software (version 1.30.2) was used to calculate the alpha-diversity indices including Sobs, Chao1, Shannon, Simpson, and coverage. Then, an independent *t*-test was employed to compare the indices between males and females. Beta-diversity analysis was performed utilizing non-metric multidimensional scaling (NMDS) based on the Bray–Curtis distance calculated from OTU data. Furthermore, permutational multivariate analysis of variance (PMANOVA) (Adonis test with 9999 permutations) was conducted to evaluate the differences between the intestinal bacterial communities of the two sexes. Comparisons of taxon abundances at the phylum and genus levels between the two sexes were performed via independent *t*-test. The linear discriminant analysis effect size (LEfSe) was utilized to identify distinct biomarkers associated with sex [33]. The prediction of microbial functions was achieved through the phylogenetic investigation of communities via the reconstruction of unobserved states (PICRUSt2) using high-quality sequences. An independent *t*-test was conducted to investigate the mean difference between two sexes. The data were analyzed using R 3.3.1 version on the online platform of Majorbio Cloud Platform. The resulting figures were generated using Origin (version 2019).

## 3. Results

### 3.1. Bacterial Sequence Data

**Figure 1:**
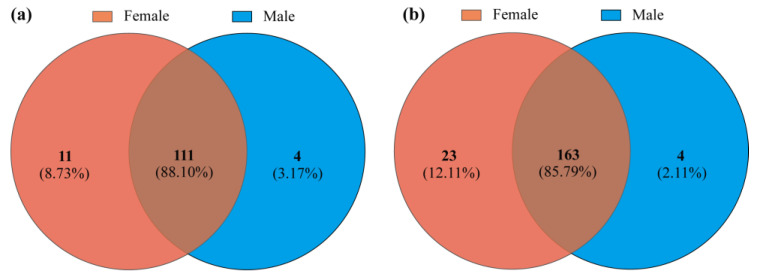
Venn diagram based on the genera (**a**) and species (**b**) of intestinal bacteria from two sexes of *Pardosa astrigera*.

A total of 284,937 original sequencing reads were generated from all samples. After quality filtering, 177,216 valid reads were obtained, with an average of 29,536 valid reads per sample. The estimated coverage values for all reads exceeded 99%, indicating that the existing sequences fully covered the diversity of the bacterial community samples (Table 1). Approximately 226 OTUs were detected in all samples and clustered at a sequence similarity of 97%. A total of 126 genera and 190 species were identified, of which 111 (88.10%) genera and 163 (85.79%) species were shared by both sexes, with 11 genera exclusive to females and 4 genera exclusive to males (Figure 1).

### 3.2. Intestinal Bacterial Diversity

The results of the independent *t*-test showed significant variations in bacterial alpha-diversity between two sexes of *P*. *astrigera* (Table 1). Among the four alpha-diversity indices, the Chao1 index (*p* < 0.05) and Shannon index (*p* < 0.01), were significantly higher in females than in males, the Simpson index (*p* < 0.01) was significantly lower in females than in males, while the Sobs index showed no significant difference between the two sexes (*p* > 0.05).

**Figure 2:**
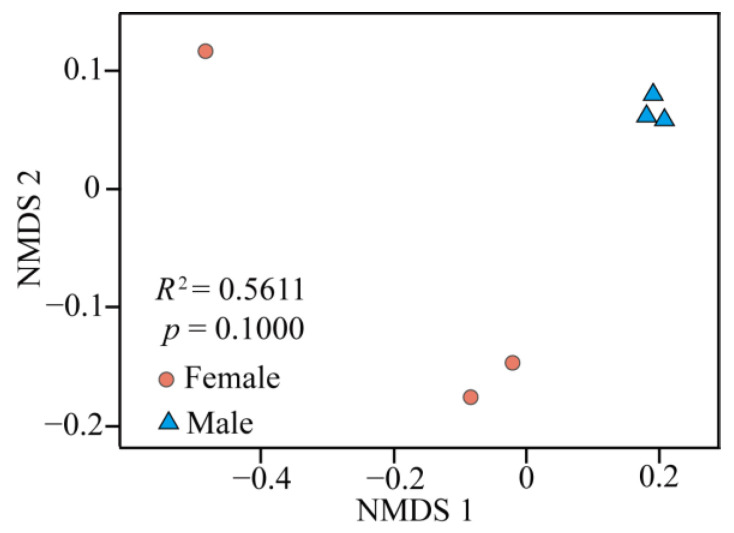
Non-metric multidimensional scaling (NMDS) for the visualization of intestinal bacterial community dissimilarities between the two sexes of *Pardosa astrigera* based on OTU data.

Non-metric multidimensional scaling (NMDS) based on the detected OTUs across the samples indicated that the male bacterial communities were well separated from those of females, even though the results of the permutational multivariate analysis of variance showed that there was no significant difference between the two sexes (*p* = 0.1000, *R*^2^ = 0.56; Figure 2). Moreover, the samples from the males were closely clustered, while those from the females were rather dispersed.

### 3.3. Intestinal Bacterial Community Structure

**Figure 3:**
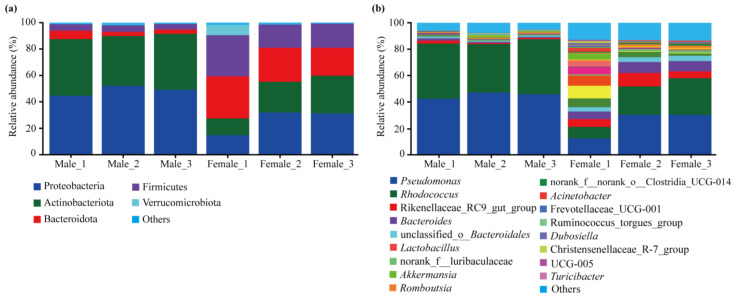
The intestinal bacterial compositions at the phylum (**a**) and genus levels (**b**) of *Pardosa astrigera*. Taxa with less than 1% membership in the samples of each sex are grouped within “Others”.

**Figure 4:**
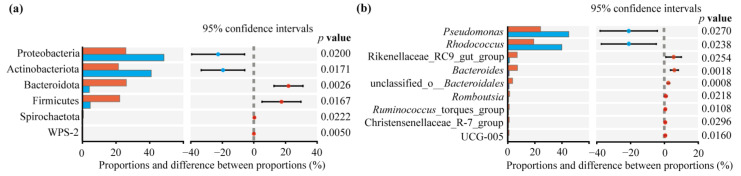
The intestinal bacterial compositions and differences at the phylum (**a**) and genus levels (**b**) in two sexes of *Pardosa astrigera*. Red represent the females and blue represent the males. Differences based on the independent *t*-test and a two-tailed *p* values less than 0.05 were considered significant (with bootstrap values of 95%). *p* < 0.05 indicates significant difference.

The relative abundance of dominant intestinal bacteria (>1%) showed sex differences at the level of different taxa (Figure 3). At the phylum level, a total of 17 phyla were identified, among which Proteobacteria, Actinobacteriota, Bacteroidota, and Firmicutes were the dominant phyla in both sexes (Figure 3a). At the genus level, a total of 126 genera were found from the data, among which 16 dominant genera were found in females, and six dominant genera were found in males (Figure 3b). The results of the independent *t*-test showed that the relative abundances of *Pseudomonas* (belonging to Proteobacteria) and *Rhodococcus* (belonging to Actinobacteriota) in males were significantly higher than in females (*p* < 0.05), while these of the other seven dominant genera, including *Rikenellaceae* RC9 gut group and *Bacteroides* (all belonging to Bacteroidota), were significantly higher in females than in males (*p* < 0.05, Figure 4b). No significant differences (*p* > 0.05) were found in the dominant genera between the two sexes except for those shown in Figure 3.

**Figure 5:**
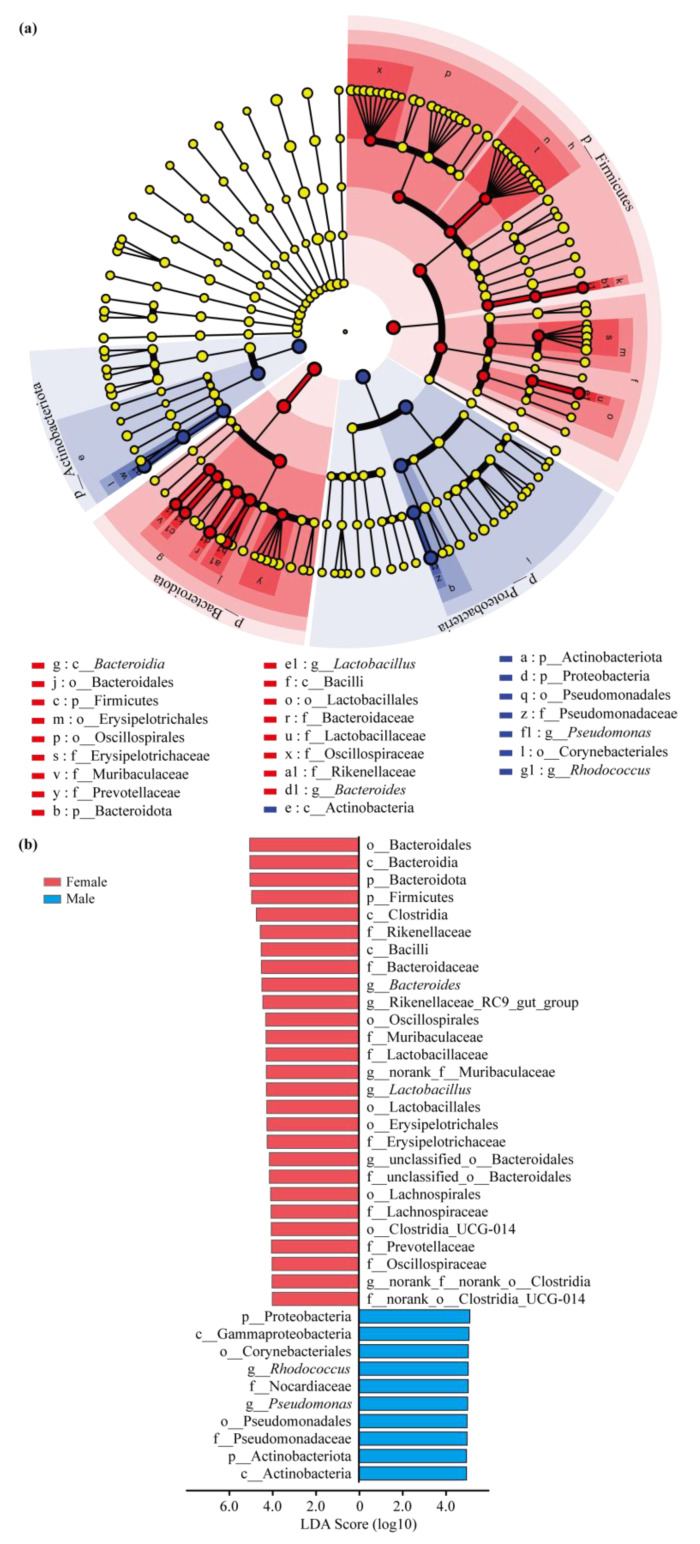
Cladogram of bacterial biomarkers in two sexes of *Pardosa astrigera* based on the LEfSe analysis. (**a**) Cladogram showing the relationships among taxa (from the inner to outer rings, phylum, class, order, family, and genus). Female-enriched taxa (red dots), male-enriched taxa (blue dots), and taxa enriched closely in two sexes (yellow dots). (**b**) Bar plot showing the different taxa with an LDA score > 4 and *p* < 0.05.

Consistent with the community composition, the bacterial communities between females and males were significantly different according to the results of the LEfSe analysis of the relative abundance of bacterial biomarkers. Six genera, including *Bacteroides* (from phylum to genus) and *Lactobacillus* (from phylum to genus), were significantly enriched in females, and *Pseudomonas* (from order to genus) and *Rhodococcus* (from phylum to genus) were significantly enriched in males (LDA score > 4, *p* < 0.05; Figure 5a,b).

### 3.4. Functional Predictions with PICRUSt2

**Figure 6:**
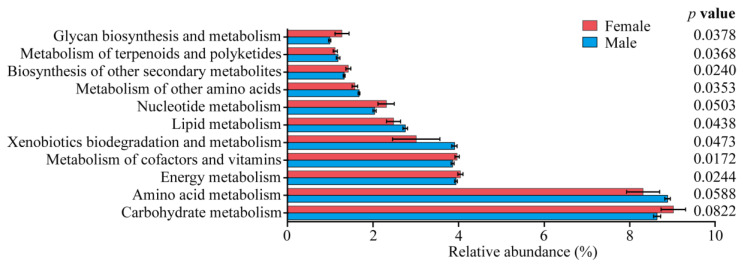
Comparison of the predicted functions of the intestinal bacteria of *Pardosa astrigera*. The difference based on an independent sample *t*-test. *p* < 0.05 indicates significant difference.

The results of the functional predictions of intestinal bacteria using PICRUSt2 analysis identified 11 level-2 pathways related to key metabolic functions (Figure 6). Among them, glycan biosynthesis and metabolism, the biosynthesis of other secondary metabolites, the metabolism of cofactors and vitamins, and the energy metabolism were significantly higher in females than in males (*p* < 0.05), while the metabolism of terpenoids and polyketides, the metabolism of other amino acids, the lipid metabolism, and the xenobiotics biodegradation and metabolism were significantly lower in females than in males (*p* < 0.05).

## 4. Discussion

The proportions of intestinal bacteria shared by male and female *P*. *astrigera* during overwintering are very high both in both the genus and species levels. Surprisingly, there were significant differences in α-diversity indices, the relative abundance of the dominant phyla and genera, and the predicted functions of intestinal bacteria between males and females. Our results demonstrate that there are sex-specific differences in the intestinal bacterial communities of the overwintering *P*. *astrigera*.

### 4.1. Intestinal Bacterial Diversity

Consistent with our first hypothesis, we found that female spiders had higher intestinal bacterial richness than male spiders, even though all spider individuals were collected from a very limited spatial range with a highly homogeneous environment, and the females were usually found together with the males. There were very high levels of shared species (85.79%) among the two sexes, which mean that the environmental factors (including prey) that had significant impacts on intestinal bacteria were rather similar in both sexes. Therefore, we speculate that the differences in intestinal bacterial communities between female and male samples may relate to their diverse physiological needs according to sex. Sexual variation has been proven to be a crucial factor in shaping the intestinal bacterial community in spiders, beetles, and mosquitoes, and the more active females have been found to be associated with a higher intestinal microbiota diversity [23,24,25,26]. Interestingly, Gao et al. [23], who collected spiders from exactly the same place and used identical methods to ours, detected a lower intestinal bacterial diversity in female *P*. *astrigera* during the mating season in spring. Female *P*. *astrigera* adopt a “sit and wait” strategy during their reproductive stage [34], while they are more active in hunting prey before overwintering to obtain enough energy for cold resistance and subsequent reproduction. Furthermore, the β-diversity visualization using NMDS also illustrated that the β-diversity of females was also higher than that of males, though there were no significant differences between two sexes.

Compared to the results reported by Gao et al. [23], the present study indicated a higher intestinal bacterial diversity of *P*. *astrigera* in winter than in the early spring season. In line with previous studies, our findings indicate the importance of the environment to the diversity of intestinal bacteria [8]. In the early spring season, the environment in which spiders live is extremely simple, with only some corn straw from the last year left on exposed land. However, various weeds and leaf litter on the ground form an inhomogeneous habitat before winter. Notably, the Shannon index of the female *P*. *astrigera* in the winter was more than three times higher, while the Sobs and Chao1 indices were almost two times higher, than in the early spring. In addition to environmental factors, female spiders are more active in autumn; however, the lower level of activity in spring is also an important factor.

### 4.2. Intestinal Bacterial Community Structure

As expected, for our first hypothesis, we found significant differences in the relative abundance of the intestinal bacteria in females and males at both the phylum and genus levels, according to our comparison of compositions and the LEfSe analysis. For example, only two phyla (Proteobacteria and Actinobacteriota) and two genera (*Pseudomonas* and *Rhodococcus*) were significantly higher in males than in females, whereas all the other dominant phyla and genera (Figure 4) were significantly higher in females than in males. At the species level, the two most abundant species of *Pseudomonas versuta* (Proteobacteria) and *Rhodococcus erythropolis* (Actinobacteriota) contributed 85.22% (45.18% and 40.04%, respectively) to the bacterial community in males, whereas they contributed 43.68% (24.38% and 19.30%, respectively) to females (Table S1). As a result, the Simpson index in males was more than three times higher than in females, although the shared species was as high 85.79% in both sexes. Moreover, five dominant genera (*Rhodococcus*, *Lactobacillus, norank f Muribaculaceae*, *Ruminococcus,* and *Acinetobacter*) in this study were also found to be dominant in the early spring season in *P*. *astrigera* in Gao et al. [23]. However, more than two-thirds of the dominant genera (10 genera) found in this study are not included in the dominant genera in the result of Gao et al. [23].

### 4.3. Potential Implications of the Intestinal Bacteria in the Cold Tolerance of Spiders

Intestinal bacteria play a crucial role in understanding the survival rate of the host during overwintering by adaptation to cold tolerance, metabolism, and immunity in invertebrates [12,35,36]. Some of the dominant genera found in this study are worth paying attention to in future research on *P*. *astrigera* spiders’ cold tolerance. For example, *Pseudomonas* and *Acinetobacter* can maintain functional activity at low temperature [37,38], and some species that belong to *Pseudomonas* are ice nucleating-active microbes and can elevate the supercooling points of their host [10,39]. *Lactobacillus, Akkermansia,* and *Christensenellaceae* R-7 may help the host to maintain a healthy bowel during hibernation [40,41,42]. Moreover, the most dominant species (*Pseudomonas versuta*) in this study was first isolated from Antarctic soil [43], and the second dominant species (*Rhodococcus erythropolis*) can maintain biological activity at low temperature [44]. Thus, they may have the potential to play key roles in the cold tolerance of overwintering *P*. *astrigera*.

## 5. Conclusions

The cold tolerance of *P*. *astrigera* is a manifestation of their adaptability to changes in environmental temperature. Our results suggest that the diversity and composition of the intestinal bacteria differed between the two sexes of the overwintering *P*. *astrigera*. However, we cannot interpret the impact of the intestinal bacteria on the differences in the cold tolerance of the males and females. Our research reveals the significant role of sex in shaping the diversity and composition of intestinal bacteria in overwintering *P*. *astrigera*. In future research, we suggest that we should first focus on the functional profiles of *Pseudomonas versuta* (Proteobacteria) and *Rhodococcus erythropolis* (Actinobacteriota) on overwintering *P*. *astrigera*.

## Figures and Tables

**Table 1:** Results of independent *t*-test on intestinal bacterial alpha-diversity indices (mean ± S.D.) between two sexes of *Pardosa astrigera*.

	Sobs	Chao1	Shannon	Simpson	Coverage
Female	162.67 ± 15.95	168.33 ± 13.30	3.13 ± 0.51	0.12 ± 0.07	0.9998
Male	142.33 ± 2.52	145.23 ± 3.53	1.60 ± 0.10	0.37 ± 0.02	0.9997
*p* value *	0.0946	0.0438	0.0069	0.0036	0.3050

* *p* < 0.05 indicates significant difference.

## Data Availability

The original data on the gut microbiota relative abundance in spiders are available from the NCBI Sequence Read Archive (SRA) database (Accession number: PRJNA1102975).

## Supplementary Materials

The following supporting information can be downloaded at: https://www.mdpi.com/article/10.3390/insects15070490/s1, Table S1: Comparison of dominant species of intestinal bacteria (mean ± S.D.) between two sexes of *Pardosa astrigera* based on independent *t*-test.
