# Supplementary material for: The Potential Implications of Sex-Specific Differences in the Intestinal Bacteria of the Overwintering Wolf Spider Pardosa astrigera (Araneae: Lycosidae)
Source: Insects. 2024 Jun 30;15(7):490. doi: 10.3390/insects15070490 (PMC11276740; doi:10.3390/insects15070490)
Supplement: Supplementary file 1 [file insects-15-00490-s001.zip › insects-3021905-supplementary.pdf]

insects

MDPI

Supplementary Material

The Potential Implications of Sex-Specific Differences in the Intestinal Bacteria of the Overwintering Wolf Spider *Pardosa astrigera* (Araneae: Lycosidae)

Ningkun Li [1], Quan Yuan [1], Yaru Qi [1], Pengfeng Wu [1], Shuyan Cui [1,*], and Guo Zheng [1,2,*]

[1] College of Life Science, Shenyang Normal University, Shenyang, China
[2] Liaoning Key Laboratory for Biological Evolution and Agricultural Ecology, Shenyang 110034, Liaoning, China

* Correspondence: cui.shu.yan@163.com; zhengguo@synu.edu.cn

Table S1. Comparison of dominant species of intestinal bacteria (mean ± S.D.) between two sexes of *Pardosa astrigera* based on independent *t*-test

Phylum	Family	Species	Female (%)	Male (%)	*p* value *
Proteobacteria	Pseudomonadaceae	*Pseudomonas versuta*	24.38 ± 10.28	45.18 ± 2.40	0.0270
Actinobacteriota	Nocardiaceae	*Rhodococcus erythropolis*	19.30 ± 9.71	40.04 ± 2.87	0.0238
Bacteroidota	Rikenellaceae	uncultured_bacterium_g__Rikenellaceae_RC9_gut_group	5.96 ± 2.04	1.16 ± 0.72	0.0183
Bacteroidota	unclassified_o__Bacteroidales	unclassified_o__Bacteroidales	3.66 ± 0.40	1.05 ± 0.2	0.0008
Bacteroidota	Muribaculaceae	uncultured_bacterium_g__norank_f__Muribaculaceae	2.82 ± 4.30	0.05 ± 0.01	0.3267
Verrucomicrobiota	Akkermansiaceae	*Akkermansia muciniphila*	2.56 ± 4.44	0	0.3736
Bacteroidota	Bacteroidaceae	unclassified_g__*Bacteroides*	1.66 ± 0.64	0.30 ± 0.19	0.0239
Firmicutes	Peptostreptococcaceae	*Romboutsia ilealis*	1.49 ± 0.52	0.40 ± 0.03	0.0218
Bacteroidota	Prevotellaceae	uncultured_bacterium_g__Prevotellaceae_UCG-001	1.59 ± 2.55	0.04 ± 0.04	0.3511
Firmicutes	norank_o__Clostridia_UCG-014	unclassified_g__norank_f__norank_o__Clostridia_UCG-014	1.61 ± 2.62	0.01 ± 0.01	0.3512
Bacteroidota	Bacteroidaceae	*Bacteroides barnesiae*	1.41 ± 0.96	0.20 ± 0.11	0.0956
Firmicutes	Lachnospiraceae	uncultured_Clostridiales_bacterium_g__*Ruminococcus*_torques_group	1.18 ± 0.31	0.38 ± 0.03	0.0108

Firmicutes	Erysipelotrichaceae	uncultured_bacterium_g__*Dubosiella*	1.47 ± 2.55	0	0.3739
Firmicutes	Lactobacillaceae	*Lactobacillus reuteri*	1.31 ± 1.16	0.14 ± 0.08	0.1563
Bacteroidota	Rikenellaceae	unclassified_g__Rikenellaceae_RC9_gut_group	1.14 ± 0.71	0.22 ± 0.09	0.0887

* $p < 0.05$ indicates significant difference
